# Reprotoxic Impact of Environment, Diet, and Behavior

**DOI:** 10.3390/ijerph19031303

**Published:** 2022-01-24

**Authors:** Alessandra Gallo

**Affiliations:** Department of Biology and Evolution of Marine Organisms, Stazione Zoologica Anton Dohrn, Villa Comunale, 80121 Naples, Italy; alessandra.gallo@szn.it

**Keywords:** reproduction, nutrition, behavior, toxicity

## Abstract

Reproductive health is progressively declining due to multiples endogenous and exogenous factors, such as environmental contaminants, diet and behavior. Accumulated evidences confirm that fertility and reproductive function have been adversely affected by exposure to chemical contaminants released in the environment. Today, the impact of diet and behavior on reproductive processes is also receiving special attention from the scientific community. Indeed, a close relationship between diet and fertility has been proven. Furthermore, a combination of unhealthy behavior, such as exposure to hazardous compounds and stress factors, poses living organisms at higher risk of reprotoxic effects. In particular, it has been described that poor life behaviors are associated with reduced male and female fertility due to decreased gamete quality and function. Most of the erroneous behaviors are, furthermore, a source of oxidative stress that, leading to epigenetic alterations, results in an impaired reproductive fitness. This review reports the detrimental impact of the most common environmental chemical stressors, diet, and behavior on reproductive functionality and success. Although clear evidences are still scarce, reassuring data are provided that a healthy diet and reverting unhealthy lifestyles may be of help to recover physiological reproductive conditions.

## 1. Introduction

Over the past few years, several industrial, agricultural, and urban activities have released into the environment a huge amount of chemical contaminants, which include metals, nanoparticles, plastics, antifouling substances, and other biocides. The environment is also impacted by land erosion and global warming climatic changes [1]. Chemical pollution and thermal rise are creating increasing alarm in governments and the scientific community because they can negatively affect reproduction of living organisms [2,3,4,5]. Environmental pollution is progressively exposing animal and human reproductive organs and tissues to several different toxicants, which exert adverse effects on their morphology and functionality. In particular, ovaries and testis are the organs where oocytes and spermatozoa undergo formation, maturation, and fertilization competence acquisition. Gamete physiological dysfunctions due to environmental chemical insults have been shown to affect the reproductive outcome in terms of fertilization, embryo development, pregnancy rate, and live births [5]. To date, however, an increased concern is also related to the potential threat exerted by unhealthy diet and behavior to normal physiological and reproductive functions in males and females who undergo daily exposure to these different dangerous habits.

Reproduction is the intricate process that starts with the production of gametes and ultimately leads to the zygote formation, which is the first cell of a new developing individual. Gametogenesis is the differentiation program that occurs in male and female gonads and that transforms primordial germ cells into mature gametes competent for fertilization and includes nuclear and cytoplasmic maturation. Reciprocal gamete activation is a multistep process essential for their interaction at fertilization and the following embryo development. In particular, oocyte extracellular coats are able to transform quiescent spermatozoa into motile cells able to reach and bind the oocyte, undergo acrosome reaction, penetrate the extracellular coat, and, finally, fuse with the plasma membrane, releasing the activating factor into the oocyte cytoplasm [6,7]. Hereafter, the second step of gamete activation involves electrical, structural, and metabolic changes in the oocyte, accompanied by meiosis completion and the beginning of embryo development [7]. All these specific events are closely interconnected and strictly rely on a normal physiological status of gonads and gametes and involve different hormones. The hormonal control systems of reproduction are dynamic processes, which are vulnerable to several sources of physiological stress caused by microenvironmental disturbs [8]. A large amount of studies reveals that a healthy status of parents is an essential prerequisite to achieve successful fertilization. Unfortunately, in the modern world, a series of dangerous habits are impairing reproductive health due to unhealthy nutritional choices, weight dysfunctions, food customs, chronic and excessive smoking, drugs, and alcoholism associated with stress, infections, diseases, professional hazards, and abuse of electronic devices [9,10,11,12]. Currently, peculiar attention is paid to the association between diet and reproductive outcomes. In particular, an excessive weight gained during pregnancy places the offspring at risk for metabolic, and not metabolic, diseases, probably via epigenetic mechanisms of embryo programming [13]. To contrast the adverse effect of an unhealthy diet pattern, numerous studies are aimed at disclosing the positive impact that specific food groups and nutritional supplements exert to enhance male and female reproductive functions [14].

**Figure 1:**
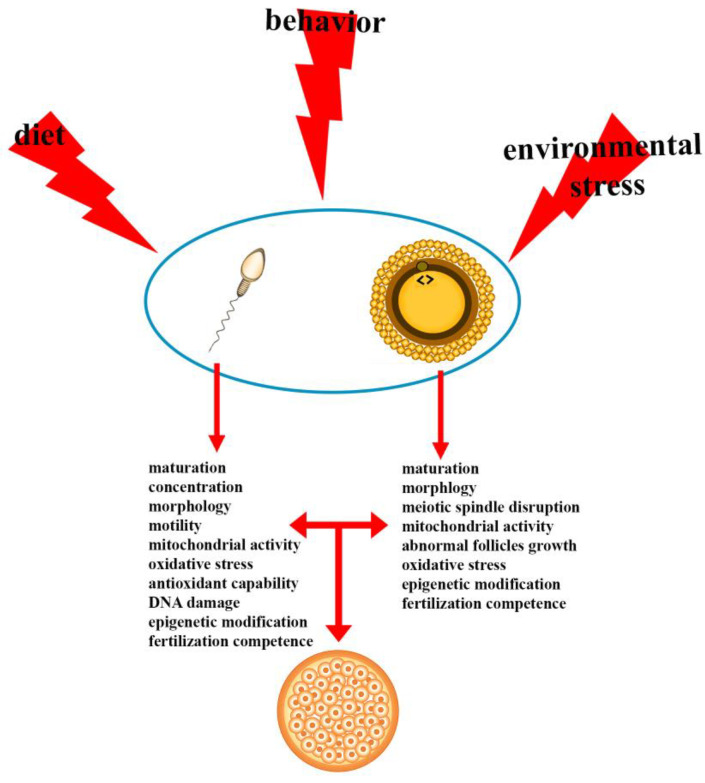
Reprotoxic impact of environment, diet, and behavior. Diet, behavior, and environmental chemical stressors affect diverse gamete quality parameters and, in turn, embryo development.

In this review, we report updated information on different impairments of male and female reproductive physiology exerted by environmental chemical stressors, diet, and behavior (Figure 1). 

## 2. Environmental Chemical Stressors

The impact of diet and behavior on reproductive health is now receiving special attention from the scientific community, which, however, has always had a special concern for the impacts of environmental contaminants on fertility and reproductive process. 

Over the last few decades, agricultural and industrial activities resulted in the release of numerous contaminants into terrestrial and marine environments. These substances, known as xenobiotics, include metals, biocides, and plastics [15]. Several xenobiotics exhibit endocrine disrupting activity (endocrine disruptors (EDs)), which interfere with the natural hormonal pathways, impairing both body organs and systems and, thus, representing a serious threat to reproductive fitness of animals and humans [16]. EDs may also affect gamete physiology and quality, resulting in a decline of fertility potential [17]. Metals are considered as major source of environmental contamination due to their toxicity and ability to bioaccumulate. Some of them, such as magnesium, calcium, manganese, and zinc, are involved in several physiological processes and are defined as essential metals, which, however, may result in being toxic at high concentrations [18]. The nonessential metals, such as lead, cadmium, and mercury, do not play any physiological role and are highly toxic for living organisms. The detrimental effects of metals are attributable to diverse cellular and biochemical processes in living organisms. Indeed, exposure to different metals has been demonstrated to induce oxidative stress, which, in spermatozoa, is known to generate harmful plasma membrane lipid peroxidation [19,20]. Indeed, in the freshwater crab, it has been evidenced that lead exposure is able to reduce sperm plasma and acrosome membrane integrity through oxidative stress generation [21]. In mammalian models, such as mice, lead affects the main functional sperm parameters, altering the male fertility competence [22]. Cadmium is one of the most toxic metals due to its long half-life and widespread occurrence. Due to its low excretion rate and its accumulation in reproductive organs, it has been shown to decrease fertilization rate and reproductive outcome by affecting sperm quality and functionality [23,24,25,26]. Protamine-like proteins (PLP) are sperm nuclear basic proteins, which play a role in chromatin condensation during spermiogenesis. Parental short exposure of *Mytilus galloprovincialis* to cadmium has been demonstrated to alter PLP properties, resulting in modifications of chromatin organization of spermatozoa that are essential for the success of fertilization [27]. Furthermore, cadmium bioaccumulation has been observed in the testis of mussels housed at a marine polluted site and correlated with PLP alteration form characterized by a decrease of DNA binding ability [28]. Cadmium in a dose-dependent manner induces oxidative damages and heat shock protein expression, even in toxi-tolerant liverwort species [29]. In the ascidian *Ciona robusta*, cadmium exposure affects the electrical proprieties of oocyte plasma membrane and embryo development [30,31,32,33]. Chronic exposure to cadmium has been demonstrated to affect sperm quality, oocyte size and to impair fertilization and embryo morphology in sea urchin [34,35]. On the other hand, oocytes of different marine species have been reported to tolerate cadmium exposure [36], whereas cumulus-oocyte complexes exposed to cadmium resulted in impaired oocyte fertilization in livestock animals [37]. In the same species, recent studies have evidenced that reproductive activity has been also been affected by mercury exposure through the alteration of PLP proteins capacity to bind and protect DNA from oxidative damage, suggesting a possible molecular mechanism of mercury-related infertility and providing new rapid and efficient chromatin-based genotoxicity assays for environmental biomonitoring programs [38,39]. 

Biocides are used worldwide, as compounds mainly represented by pesticides, herbicides, insecticides, and antifouling paints. Reproductive effects associated with pesticide exposure are decreased fertility, developmental abnormalities, ovarian disorders, and disruption of the hormonal function from aquatic to mammal species. Moreover, a close association between pesticides and sperm quality impairment has been demonstrated for almost two decades now [40]. Herbicides used in agriculture, such as atrazine, have been classified as potent EDs since, in crayfish females, they cause a dysregulation of hormone synthesis in the ovaries, giving rise to oocyte size reduction [41]. Furthermore, recently, an important atrazine-related epigenetic alteration in oocytes and spermatozoa has been demonstrated, accompanied by developmental diseases with transgenerational epigenetic inheritance [42]. An emerging toxic herbicide is glyphosate, which is able to reduce oocyte swelling in the aquatic carp *Cyprinus carpio*; however, a less harmful impact is exerted on sperm motility and following embryo survival without generation of embryo malformations and poor larval development [43].

Antifoulings are protective paints developed to release biocides, to prevent the attachment of fouling organisms on underwater surfaces. The released biocides have been demonstrated to impair reproductive physiology of marine organisms. Tributyltin (TBT) has been proven very effective in dealing with fouling; nevertheless, due to its strong ecotoxicity and the negative ecological effects observed worldwide, it has been banned from the market since 2008. Indeed, it was the first antifouling biocide identified as ED in mollusk reproduction, inducing the male sex characteristics in females, known as imposex, acting on aromatase inhibition, which, in turn, leads to increased testosterone levels and decreased estradiol [44,45,46]. In ascidians, TBT has been proven to impede early gamete interaction and, hence, fertilization events [47,48]. Diuron and chlorothalonil are the organic booster biocides mainly used, that have been replaced organotin compounds in antifouling paints. Nevertheless, these new generation antifoulants have also been demonstrated to negatively affect marine organism reproduction. In ascidian, the pre-exposure of oocytes to diuron and chlorothalonil affects the electrical properties of the oocyte plasma membrane and causes damage to the offspring by inducing larval malformation [49]. Furthermore, diuron has been demonstrated to exert genotoxic effects on oyster gametes, without a clear impairment of sperm mitochondrial activity and acrosomal function [50]. 

Plastics are synthetic polymers used in a series of commercial products, such as bags, beverage bottles, and food storage containers. During plastics manufacturing, different chemical compounds are added, such as polychlorinated biphenyls (PCBs), polycyclic aromatic hydrocarbons, phthalates, organochlorine pesticides, and bisphenol A (BPA), which can leach and act as EDs interfering with hormone systems and cause reproductive disorders. In women of childbearing age or during pregnancy, these additives may exert negative effects since they are able to cross the placenta and accumulate in the fetus [51]. Moreover, phthalates have been proven to impact, in a dose-dependent manner, sperm quality by affecting diverse parameters, such as concentration, morphology, and motility. Similarly, PCB resulted in being detrimental to sperm count and motility, exerting toxicity on reproductive processes on aquatic life, especially those of freshwater organisms [52,53,54,55]. Despite the high level of attention given to the toxic impact of BPA, only a few studies have evaluated its effect on human gamete quality [56]. Environmental exposure to BPA has been shown to affect sperm and oocyte quality through epigenetic mechanisms that represent a risk for the resultant offspring and their reproductive ability. Growing evidence exists that BPA, present in many food plastic manufacturing practices, is an ED harmful to animal models and humans, especially concerning children behavior [57]. By interfering with steroid signaling, BPA has been shown to exert adverse effects on reproductive health and fertility, couple reproductive outcome, and trans-generational impacts on both parents and the offspring [58]. Although the mechanism of toxic action of BPA has not been fully elucidated, it has been demonstrated to affect spermatogenesis and sperm quality and, in turn, reproductive potential of the offspring in males and ovaries, folliculogenesis, oocyte quality, embryo development reducing the reproductive outcome, and in vivo and in vitro fertilization in females [59,60]. Evidence are provided that, along with a threat to female fertility, the BPA’s main deleterious effects are associated with offspring development during perinatal exposure, possibly generating dysregulation of the hypothalamic-pituitary-ovarian axis, placing the following generations at risk of developing BPA-related diseases themselves [61]. Furthermore, BPA has been proven to affect female androgen receptor-mediated signaling, which regulates Kallikrein-Related serine Peptidase 3 secretion [62].

A close relationship between air pollutants and sperm quality impairment has been reported in the last few decades [54,63]. In particular, airborne particulate matters resulted in being genotoxic to male germ cells, inducing DNA damage and alterations in sperm functionality and fertilization potential [64]. The recent COVID-19 pandemic spread has added serious further concern due to the possible synergy between virus infection and other sources of pollution. Atmospheric pollutants deserve peculiar attention since producing oxidative stress and inflammation may contribute to further damages, making the organisms vulnerable to infections of pathogens and viruses [65]. Very recent studies have suggested a possible impact of COVID-19 on male fertility due to the overexpression of the alveolar angiotensin-converting enzyme 2 receptor, which is the entryway of the virus into the organism, involving lungs and testis, and the dangerous relationship with air fine particulate matter involved in dysfunctions of blood-testis barrier [66,67]. The Land of Fires is a geographic area in the south of Italy, where illegal activities in toxic waste burning are creating harmful air quality [68]. In the frame of the Ecofood fertility project, evidence have been provided for the fertility impairment of men living and working in these areas. Together with generalized fertility decline, the main damages observed arise in DNA oxidative damage, and hints are provided on a possible transgenerational inheriting of all the molecular alterations on sperm nuclear basic proteins, DNA integrity, and functional semen parameters [69,70]. Healthy young men living in Italian high-polluted areas and the south Land of Fires have been recently enrolled in a randomized controlled trial, submitting them to a specific Mediterranean diet and moderate physical activity program. This interesting survey showed that these interventions, based on targeted diet and physical activity, are able to determine a remarkable improvement of sperm quality parameters [71].

## 3. Diet and Behavior Impact on Male Reproductive Health

During spermiogenesis, the last phase of spermatogenesis, the spermatid undergoes a dramatic structural remodeling that transforms it into a mature spermatozoon consisting of the head, the neck, and the tail. Each one of these structures has a specific function for accomplishment of fertilization. In the head, a sperm nuclear compaction occurs due to the condensation of chromatin that provides the hydrodynamic structure aimed at protecting the sperm genomic integrity and improving sperm motility [72]. Together with chromatin condensation, physiological DNA fragmentation occurs to facilitate the histone/protamine transition, which, however, is promptly repaired by specific enzymes [73]. Further DNA fragmentation repair also occurs in the oocyte following fertilization, reinforcing the idea of a need for genomic integrity in successful reproductive events [74,75]. 

At present, however, a huge amount of studies is available on the occurrence of genomic integrity disorders following the exposure to environmental insults and on related adverse consequences on fertilization potential, embryo development, pregnancy outcome, and the offspring health [76,77]. Overlapping the nucleus, the acrosome, a small exocytotic vesicle, allows sperm penetration across the extracellular coat, whereas a mitochondrial ring, which provides the energy for the tail movement, surrounds the neck. Sperm metabolism and respiration strictly rely on oxygen utilization having, as byproducts, reactive oxygen species (ROS) formation. These play a positive role in some of the physiological processes of spermatozoa, such as capacitation, acrosome reaction, and fusion of gametes plasma membrane [78]. However, in high quantity, ROS are involved in lipid peroxidation of plasma membrane, generation of oxidative stress, and related cytotoxicity and DNA damage. Recent literature reports increasing evidence that ROS and oxidative stress are intermediate modulators between poor quality of life and the impact on male fertility [79,80].

Male factors are responsible for approximately 50% of couple subfertility all over the world [81]. Therefore, the first diagnostic approach in the clinical screening is the spermiogram, which evaluates different sperm parameters, such as concentration, motility, and morphology, according to WHO guidelines (WHO, 2010). A recent evidence-based meta-analyses reported a warring 57% diminution in mean sperm concentration in European, American, Asian, and African males, over the past 50 years [82,83]. 

Adhesion to healthy diet, maintenance of ideal weight, and exercise may positively affect fertility for both men and women. In particular, dietary pattern, including fish, vegetables, fruits, poultry, whole grains, and healthy fats, have been related to fertility improvement and pregnancy outcome [14,84].

On the contrary, unhealthy diet may lead to being overweight, obese, and diabetic, which are increasing diseases in the western world due to abnormal or excessive fat accumulation in adipose tissues that, in turn, generates changes in hormone levels.

Besides environmental and genetic cues, erroneous behaviors appear to be a major cause of obesity. Many epidemiological studies report a significant association between being overweight/obese and male reproductive disorders possibly due to secondary hypogonadism resulting in the significant decline in testosterone production [85,86]. Other than affecting the complex interplay of the pituitary-gonads axis and different sexual functions, obesity exerts adverse effects on sperm parameters modulated by an indirect Sertoli cells dysfunctions, increased scrotal temperature associated with oxidative stress but all over the increased liposolubility of environmental toxicants in fat tissues. In fact, an inverse correlation of waist circumference and sperm count and motility has been demonstrated, together with an alteration in both mitochondrial activity, DNA integrity and the increased aneuploidy for triple 21 chromosomes. Although the mechanism of action of this negative impact is far to be elucidated, a confirmation of this cause-effect process comes also from a reduced in vitro fertilization success, pregnancy rate and an increased pregnancy loss involving overweight and obese men [87]. Bad eating habits are often causes of diabetes, which is a widespread and highly disabling disease often associated with obesity. A relationship between diabetes and reproduction has been debated for a while and, apart a relationship with erectile dysfunctions and retrograde ejaculation, few studies were aimed at correlating diabetes and insulin dependence to male infertility and altered sperm parameters [88]. Over the last decade, some hints appeared showing an impact of male diabetes on molecular changes underling sperm quality and function [89]. In particular, sperm DNA damage was reported to be associated with miscarriage increase, whereas an interesting survey on 3000 couples did not evidence differences in fertilization or embryo quality but only a significantly reduced pregnancy rate, defining such reproductive difficulties as “unexplained infertility” of diabetic males [90].

Training and exercise in moderate amounts are beneficial to a series of physiological functions and general health. On the contrary, long-term intensity and duration of exercise training may cause health and reproductive problems [91]. A reduction of endocrine and spermatogenic functions has been reported after high-intensity training due to an impairment of hypothalamic-pituitary-gonadal axis and tissue inflammation in the testis, also associated with a decrease in antioxidant physiological capability. Excessive training pressure results in what was defined the “exercise-hypogonadal male condition” often associated with decreased concentrations of total and free testosterone, as well as reduced FSH and LH pulsatile secretion. Disturbances of testosterone secretion and profile strongly affect the homeostasis of reproductive system and has been observed in a series of athletes submitted to hard physical exercise, such as high-altitude marathon runners, and in professional cyclists, due to increased scrotal temperature [92,93]. More recently, it has also been suggested that excessive sports practice may worsen the clinical varicocele, which, in turn, reduces the sperm parameters quality [94]. Nevertheless, contrasting data exist on the real impact of exercise on male fertility in terms of sperm production and parameters with a trend in favor of no detrimental effect of physical activity on male fertility [95]. In fact, some studies correlated intensive exercise with worsening sperm morphology and DNA integrity, impaired spermatogenesis, and reduced seminal antioxidant capability [96]; however, more recent investigations concluded that slight training favors improvements in sperm DNA integrity and semen quality and reduces expression of inflammation markers and oxidative stress in the seminal plasma [97]. If excessive exercise is proved to be detrimental to fertility, it has been predicted that sedentary life, considered as the time spent seated, should exert an adverse effect on sperm production [98]. Later on, the same author suggested that sedentary habits may even affect fertility of the offspring [99].

Several studies on animal models exposed to nicotine demonstrated a significant decrease in sperm count and motility, DNA damage, spermatogenesis, and sperm maturation impairments, other than testicular cytotoxicity. Comparative studies on smoking and non-smoking males showed a significant association between tobacco smoking and lowered sperm volume, count, viability, and motility, associated with prevalent sperm morphological abnormalities in smokers and also related to dose, time, and duration of exposure [84,100,101]. In heavy smoker males, a generalized decrease of sperm quality, as well as damages of sperm tail and alteration of processes, such as acrosome reaction, capacitation, and sperm binding to the zona pellucida, have been reported [102,103]. Furthermore, among the many toxicants contained in cigarettes, high cadmium levels have been strictly related to sperm oxidative DNA damage, impaired fertility, and longer time to achieve pregnancy [93]. Although marijuana smoking exerts a slight deleterious effect on testicular functions [104], endocannabinoids, which are prevalent components in marijuana, have been shown to inhibit sperm motility, capacitation, and mitochondrial activity by also inducing a precocious acrosome reaction, which renders natural fertilization impossible [105]. Voluntary exposure to opiate consumption, also in view of it analgesic properties, revealed it harmful action on sperm functionality and impairment of the antioxidant ability of seminal plasma and nuclear genomic integrity [106,107]. Heroin and cocaine are drugs in which recreational use is widespread in the new generations. Chronic heroin use in males has been recently demonstrated to alter semen parameters and, in particular, the histone/protamine ratios after spermiogenesis, causing a nuclear chromatin decondensation; furthermore, heroin addiction has been also related to harmful changes of sperm pH and related to sperm motility [108]. Apart from some recent investigations on animal models reporting an association between cocaine intake and testicular toxicity [109], in humans, on the contrary, there are indications that cocaine use slightly compromises physiological spermatogenesis, without any ascertained data [84].

Alcoholism is a kind of nutritional habit included among the most common psychiatric disorders dramatically affecting public health and safety. Although many studies are aimed at experimenting with new treatments for alcohol-related diseases [110], only a few investigations still exist on how its abuse affects reproductive events. Among them, alcohol abuse has been observed to affect diverse sperm physiological parameters, such as volume, concentration, motility, and morphology; furthermore, it is also associated with apoptosis-induced sperm genomic disorders, such as DNA fragmentation, influencing fertilization, blastocyst formation rate, and increased risk of spontaneous abortion [111,112,113]. 

Nonetheless, in a systematic review aimed at correlating alcohol intake and male fertility, discriminating between abstainer/occasional, moderate, and high chronic alcohol drinkers has been suggested, since their effects appear to be different. In fact, in both animal models and humans, it has been shown that only high daily consumption of alcohol affects sperm motility, morphology, nuclear maturity, and genome integrity [114,115]. In addition, an interesting synergic effect of heavy smoking and drinking has been associated with a decline in seminal parameters [116]. In particular, a recent prospective case-control study discloses that smoking has a negative effect on sperm concentration and motility, whereas alcohol consumption mainly affects semen volume, sperm concentration, and morphology [117]. 

Electromagnetic radiation (EMR) represents another warring health hazard of the modern world, especially due to the widespread use of mobile phones, laptops, and wireless technology. It has been ascertained that long-term exposure to electromagnetic fields may alter the normal physiology of male reproductive organs and induce genotoxicity and oxidative stress that, in turn, seriously affects sperm parameters underlying fertilization, such as motility, morphology, and oocyte binding capability [118,119]. A pilot study was conducted approximately 15 years ago that demonstrated that a continuous exposure to radiation resulted in a deterioration of male gamete quality, in both animal models and humans. In the latter, increased ROS levels associated with decreased antioxidant capacity has been suggested to be responsible for DNA base adduct formation and DNA fragmentation [119,120,121,122,123]. Recent studies on animals and humans have demonstrated that radiations generate sperm tail microtubules disaggregation, mitochondria disruption, defected DNA, and impairment of gonadal tissues, such as Sertoli, Leydig, and germ cells, which, in turn, alter the androgen’s production and related hormonal pathway [80,124]. The relationship between psychological stress and infertility is a matter of concern and debate since they appear to be reciprocally influenced. Infertile couples, especially after repetitive IVF treatment and failures, may develop high psychological disorders, such as depression, anger, and frustration [125], which, in turn, may be responsible for generating further infertility problems [126]. An old, prospective study of 157 volunteers demonstrated no relationship between job and life-event stress and sperm concentration, motility, and morphometry but only a reversible fecundity diminution for stress related to the occurrence of a family members’ death [127]. A link between psychosocial stress and semen parameters has been hypothesized to be due to reduction of luteinizing hormones and testosterone pulsing, which, in turn, affect spermatogenesis and sperm quality. Discriminating between isolated and/or simultaneous jobs, as well as life and social stressful events, researchers found, in both, a negative impact on sperm quality [128]. However, unclear results were obtained for associating stress to natural disasters or wars due to different personal perceptions of such stressful conditions. Paradoxically, fertility treatments appear to be stressful events further affecting male fertility. As an example, the production of a semen sample for sperm analysis has been reported to decrease 39% of sperm concentration, and 48% of sperm motility, with a further worsening of the sperm parameters on the day of oocyte collection for IVF and ICSI. In addition, forced sexual intercourses aimed at targeted fertilization seem to also negatively influence sperm quality [129]. The decline of fertility due to psychological stress may be a transient condition since psychological interventions in infertile couples have been shown to significantly increase pregnancy rates [126]; however, psychological stress, being a very personally perceived condition, is an issue difficult to correlate with reduced fertility [95]. Job occupation is another critical source of fertility impairment. Chronic exposure to chemicals and pesticides of workers in industry and agriculture and/or of reproductive organs to high temperature coming from machinery, ovens, and saunas have been shown to strongly affect fertility parameters in both men and females [130,131]. In particular, scrotal heating generated by erroneous posture during sleeping, sedentary position in long-lasting sitting of taxi/bus drivers, and the deleterious habit of exposing the testicles to the warm battery of a laptop put on the legs may affect testicular function and is related to a decrease in sperm motility and increase in DNA fragmentation [92,132,133].

## 4. Diet and Behavior Impact on Female Reproductive Health

Quality of the parental diet is considered of primary importance in female reproductive success for the appropriate allocation of macromolecules into the oocytes. Indeed, the dietary effect on output performance in terms of egg quality and quantity is well documented. However, if studies on the reprotoxic effects of diet and behavior in males are possible by the easy provision of sperm samples, it is not the same for females due to the difficulty and ethical rules of obtaining oocytes for experimental investigation in humans, but even in other mammals. Therefore, most of the information on reproductive disorders and fertility parameters in women, such as polycystic ovary syndrome, endometriosis, uterine fibroids, fertility treatment, and pregnancy outcome, are provided by clinical trials and retrospective studies.

Recently, an interesting investigation has suggested a correlation between changing lifestyle factors, such as diet, weight, exercise, and psychological stress, and female anovulatory disorders, classified by WHO into three categories: hypothalamic-pituitary failure, hypothalamic-pituitary dysregulation, and ovarian failure [134]. The role of correct nutrition for women in reproductive age has been recently stressed since obesity has a stronger impact on female fertility than on men fertility due to the higher repercussion on perinatal morbidity and mortality [109,135,136]. Because of the excess of free fatty acids in reproductive tissues and organs, obese women are more prone to ovulatory dysfunction, increased polycystic ovarian syndrome, anovulation, reduced fecundity, poorer IVF success, early pregnancy complications and loss, fetal congenital anomalies and deaths, and generalized delayed time to pregnancy [137]. Recently, an observational study aimed at correlating body mass index and embryo competence and/or endometrial receptivity has confirmed that the maternal body being overweight is responsible for higher miscarriage rate, suggesting a possible lipotoxic mechanism exerted by incorrect body fat mass and distribution [138]. Obesity seems also to affect the oocyte and the preimplantation embryos, due to disruption of meiotic spindle and mitochondrial activities [139,140]. In men, intensive exercise is associated with hypothalamic dysfunction, leading to a disturbance, or even suppression, of GnRH pulsatility and disruption of menstrual dysfunctions [141]. Surprisingly, the maternal body being underweight, although rare in developed countries, creates a poor nutritional environment in utero and, in contrast with the excessive postnatal food supply, seems to generate a mechanism for developing chronic diseases during the offspring’s life. On the other hand, body weight in young women appears to be predictor of future fecundity, suggesting that nutrition in adolescence plays a role in the programming of the reproductive axis [142]. Therefore, to avoid widespread decline in fertility and related repercussions on descendants, well-balanced diets and nutritional habits are advisable, to maintain a healthy weight that may enhance the fertility potential in both sexes [13]. 

Female smoking has been shown to be toxic to folliculogenesis and human oocyte quality, generating abnormal follicles growth and loss, and impaired oocyte maturation and morphology, which may also affect the long-term fertility of female progeny [143]. Recent studies have provided evidence of a potential causal association between cigarette smoking and increased risk of developing polycystic ovary syndrome [144] and delayed blastulation in PMA treatments [145]. Few studies still exist on clinical implications of drug use due to limited sample size and a missing follow up. However, the presence of endocannabinoids in follicular fluids of cannabis consumers has been shown to alter ovarian and developing oocytes [146], while heroin use has been associated with placental abruption [128]. Alcohol consumption in females may be cause of subfertility; however, no clear hints of this correlation exist, and contrasting data provide further confusion [147]. In any case, alcohol use has been indicated as a risk factor for endometriosis, which is an invalidating pathology for fertility and general women’s health [148]. Inconclusive and contrasting results arise on the relationship between electromagnetic fields from mobile phones and female reproductive outcomes, with the exceptions of reported endometrial apoptosis, fetal and neonatal heart rate, and cardiac output impairment [149,150]. However, studies on animals and humans provided indications of an alteration in granulosa cells, ovarian follicle numbers, endometrial tissue, and oocyte and embryo quality [151].

The association between emotional stress and reproduction is a difficult matter to demonstrate. In women, psychological stress acts at the ovary, follicle, and oocyte level. In particular, the stress-induced cortisol increase associated with estradiol reduction seems to affect granulosa cell functions in the follicle, deteriorating oocyte quality. Oxidative stress also affects several physiological processes of female reproduction, from oocyte maturation to fertilization, embryo development, pregnancy and normal parturition, and in initiation of preterm labor [152]. Furthermore, negative pressure in lifestyle changes may generate ROS in the ovaries, in which accumulation leads to oxidative stress and, in turn, apoptosis in germ cells and in ovulated oocytes [153]. Among the few investigations on the relationship between stress and female reproduction, it has been disclosed that worse psychological conditions are mainly based on personal perception [154] and mostly attributed to infertile women struggling with their infertility [155,156]. However, the impact of occupational pressure-related psychological stress has been associated with a decline in female fertility potential and conceiving difficulties [157].

## 5. Epigenetic Regulation of Lifestyle-Affected Human Fertility

Epigenetics is a physiological process that modifies a cell phenotype without affecting the genotype. Epigenetic modifications, generally defined as “epigenome”, include DNA methylation, histone modifications, and small non-coding RNA expression, which, in turn, modulate the transcription and gene expression. Epigenetics have been shown to play a role in germ line development and affect normal embryo development by acting on meiosis, gene expression, and genomic imprinting. 

During gametogenesis, DNA methylation provides remarkable gene regulation reorganization. In case of spermatogenesis, this is a process maintained throughout post-puberty, and even during epididymis maturation. These dynamics confer to spermatogenesis a potential instability and vulnerability that may be transmitted to future generations, with serious implications on both health and reproduction [158]. Mounting evidence provided that epigenetic action is influenced by environmental factors, contributing to anomalies in the phenotypes that render individuals more vulnerable to diseases along the course of their life. In particular, parental environmental alterations have been proven to affect the phenotypes of offspring through gamete epigenetic alterations [159].

Several recent studies on epigenetic modifications demonstrate that some of the aforementioned unhealthy lifestyle habits, such as diet, alcoholism, and smoking, may generate alterations in DNA methylation, affecting, in turn, reproductive potential of males, their offspring, and grand offspring, via epigenetic-induced spermatogenesis failure and sperm alterations [160,161]. Male obesity, diabetes, and related metabolic disorders seem to generate epigenetic changes, influencing sperm function and male fertility; in particular, an association between DNA methylation, non-coding RNA modification, and the increased body mass index with a warring inherited transmission across generations has been reported. Very recent studies also evidenced an association between male obesity and altered sperm DNA methylation profiles, potentially affecting reprogramming sperm fidelity and influencing spermatogonia physiology [162]. In particular, diabetes may induce epigenetic dysregulation during spermatogenesis, which may be passed to more than one generation, placing offspring at the risk of diabetes [163]. On the contrary, maternal obesity and diabetes during pregnancy have been demonstrated to perturb the offspring epigenome, resulting in an impairment of growth and organ development, and in possible cardiometabolic disease progression [164]. Interestingly, new hints are provided that obesity and related comorbidities inherited across generations through non-genetic mechanisms are strongly tied to the epigenetic modification of gametes [86,165]. In fact, it has been suggested that some paternally-acquired environmental information can be memorized in the spermatozoa in the form of epigenetic information [166,167]. Although the two sexes follow distinct paths of epigenetic events, and gametes possess specific epigenomes, some unhealthy parental habits have been found to affect future generations’ development, via epigenetic alterations. In fact, parents being overweight/obese affects epigenetic markers in both gametes, possibly influencing epigenetic programming during embryogenesis [168]. For instance, it has been shown that lifestyles, including, nutrition, physical activity, alcoholism, cocaine use, and nicotine exposure alter reproductive function, germline epigenome/transcriptome and germline integrity as cell count, morphology, sperm motility, genomic integrity, and general structural degradation in both males and females. Because of these overall fertility damages, multigenerational and transgenerational inheritance, as well as descendants’ health impairment, occur [161,169,170]. Recently, the warring effect of epigenetic programming disruption has been demonstrated for maternal cannabis consumption exposure during critical periods of fetal development. It has been evidenced that some alterations through epigenetic mechanisms are critical for brain development and potentially associated with a series of serious offspring psychiatric disorders and/or mental illnesses [171]. Several epigenetic modifications also occur in animal and human cells following BPA exposure. The latter has been shown to modify the methylation pattern of multiple genes encoding proteins involved in reproductive physiology and to exert a straight influence on the genes responsible for DNA methylation. Inheritable epigenetic changes are generated by BPA, including hormonal and morphological alterations of male reproductive organs, and even a warring BPA-related prostate cancer [172].

## 6. Resilience of Life Style Impact on Reproduction

Water, air, and soil pollution, together with climatic changes, have been shown to impact gamete quality, fertilization, and embryo and fetal development [5,173]. Due to this worldwide alarming concern, at present, all governments are initiating the adoption of green revolution interventions to decrease CO_2_ levels in the atmosphere aimed at slowing down, or even regressing, this harmful trend. On the contrary, reproductive damages induced by unhealthy habits and customs are strongly tied to the willpower that may help the individuals to revert their lifestyle. Resilience of modifiable lifestyle factors is generating growing interest in the scientific community. Literature reveals that resilience is possible for many of the aforementioned adverse reproductive effects. Nutrition is not always a choice, as it happens in extreme environments as aquatic bodies. In marine animals, for instance, it has been demonstrated to have either a beneficial or an unfavorable effect on the way of feeding. In the last few decades, a large body of literature has reported the insidious reprotoxic impact of a phytoplankton-based diet on marine animals. Diatoms and dinoflagellates are at the basis of the marine food chain and often bloom forming. When marine invertebrates and copepods, which are a link between primary producers and higher trophic levels, are fed on mixed diatom and dinoflagellates diets, they undergo reproductive failures in terms of oocyte maturation, sperm motility, fertilization, embryo development, malformed hatching, and larval survival [174,175,176,177,178]. Otherwise, a recent example of growth-promoting effects of fucoidan dietary supplementation has been highlighted in aquatic organisms, livestock, and humans [179]. A nutritious/well-balanced diet and maintenance of a healthy weight have been shown to lower the risk of infertility in males and females [180,181]. Weight loss and improved insulin sensitivity in obese women may induce metabolic improvement, significant ovulation resumption, and reduce perinatal risks, and, interestingly, the combination with bariatric surgery has been suggested to enhance these potential benefits [182]. 

In obese people planning a pregnancy, exercise, diet, and even surgery-induced weight loss, advisable to avoid undesirable outcomes in offspring, have been shown to improve implantation rates and embryo and fetal development, suggesting that these interventions may improve offspring health [168,183,184]. In addition, obesity-related epigenetic changes in spermatozoa have been shown to perhaps be reversible through weight loss that, by improving male metabolic health, is able to increase the amount of spermatozoa with a healthy methylome, without affecting normal embryonic and fetal development [162].

Although the precise mechanism is not yet elucidated, it appears that visceral fat reduction exerts a positive impact on sex hormones serum levels and that reduction of scrotal temperature and seminal glands inflammation may further act as modulators between gonadal hormone levels and improved semen quality [87]. 

A healthier lifestyle is also beneficial in obese women since appropriate diet and exercise during the 3–6 months before fertility treatments result in improved reproductive outcomes and possible benefits for both the mother’s and the newborn’s quality life [135]. Besides no evidence has been found that weight loss close to the time of conception may reverse the obesity-related reproductive dysfunctions in both sexes [10]. Contrasting results are also provided in obese women with polycystic ovary syndrome that, after bariatric surgery intervention, showed high fertility and pregnancy rates, together with a few maternal and neonatal complications [185]. On the contrary, meta-analyses of Cochrane Central Register of Controlled Trials reported no clear effects of lifestyle intervention on menstrual regularity, miscarriage, and live birth [186]. Interestingly, the possibility to repair damages exerted by EMR mobile phones and Wi-Fi long-term exposure to male reproductive organs and gametes has been shown; in fact, EMR impacts appear to be avoidable if low-intensity, short-term, and/or intermittent exposure are adopted, with diminished risk for fertility [187]. 

Psychological stress may be a modifiable or reversible factor. A cross-sectional study also identified resilience in infertility-related stress in women with poor quality of life. It has been indicated that infertile women’s psychological status may be addressed for adequate resilience-based interventions, such as mindfulness-based skills, counseling, and even Yoga, which might potentiate and improve fertility status [188]. Most of the unhealthy habits are modifiable factors and, although solid evidence in interventional resilience effect is still lacking, simple changes in routine life customs appear to resume and ameliorate impaired fertility. Among them are included: moderate exercise load, healthy dietary pattern, reduced cell phones use and television watching, avoiding of tight underwear, and reduced or cessation of alcohol, tobacco, and drug use [95,189]. Interestingly, most of the reproductive pathologies induced by endogenous and exogenous factors are modifiable simply by relieving oxidative stress through adoption of easy lifestyle and nutritional interventions [79]. 

Pharmacological supports are also helpful for infertility reversibility since only limited endogenous factors exist to reverse these damages. In mammals, the antioxidant action of resveratrol has been demonstrated to revert spermatogenic dysfunctions exerted by high-intensity exercise [190]. The role of antioxidants substances in reverting reproductive damages in both sexes is now well established [191,192]; therefore, correcting nutritional imbalances encourages the reduction of oxidative stress through intake of antioxidant supplementation of different vitamins combination, carnitine, N-acetyl cysteine, CoQ10, selenium, zinc, and folic acid. 

Although caution has been recommended [193], these modified nutritional habits have been shown to provide a generalized improvement of subfertility traits in women and clear improvement of seminal parameters, advanced sperm function, in vitro fertilization, and live-birth rate [194,195].

Aging is known to seriously impact fertility due to a decline and deterioration in gamete quality, increased vulnerability to oxidative stress, and reduction in antioxidant defenses [118,152,196,197,198]. Although age is not a choice nor a modifiable factor, couples should be aware that delayed childbearing must be avoided as much as possible. Pregnancy, in fact, is often postponed for social, economic, and working problems, neglecting the fact that increased parental age is a major determinant for gonadal function, reproductive hormones, gamete quality, oxidative stress, and epigenetic changes. A poor combination of these factors seems to be responsible for not only reduced conception rates but also for increasing the incidences of congenital birth and fetal defects, and even of fetal deaths [95,199].

## 7. Conclusions

This review has been aimed at describing the main negative influence of environmental chemical stressors, diet, and behavior on reproductive health. It provides insight on the detrimental impact of diverse chemical stresses and metabolic disorders, such as obesity, and excessive body fat, as well as of excessive low peripheral body fat and of excessive physical exercise, on different reproductive processes. A synergy between nutrition and different lifestyle decisions, such as smoking, alcoholism, and drug use, clearly demonstrates that these strongly contribute to subfertility, infertility, and even several inherited epigenetic disorders in the offspring. It is clear that any kind of extreme social behavior may exert a reprotoxic impact; however, the possibility that withdrawal of extreme lifestyles may exert a newly positive impact on reproductive function is reassuring.

## Data Availability

Not applicable.
